# A Giant Fibroepithelial Polyp of the Vulva

**DOI:** 10.7759/cureus.39152

**Published:** 2023-05-17

**Authors:** Mustafa Cengiz Dura, Hilal Aktürk, Gül Şüheda Sungur, Waseem O.I. Alsalamin

**Affiliations:** 1 Obstetrics and Gynaecology, Bakırköy Sadi Konuk Education and Research Hospital, İstanbul, TUR; 2 Medicine, Al-Quds University, Abudis, PSE; 3 Medicine, University of Health Sciences, İstanbul, TUR

**Keywords:** desmin positive, hormone sensitive stromal cells, benign mesenchymal lesions, vulva, fibroepithelial polyp

## Abstract

Although vulvar lesions are mostly malignant, polyps represent one of the most frequent benign tumors of the vulva, typically measuring less than 5 cm in size. Larger lesions are uncommon and are likely the result of mesenchymal cell growth in the hormonally responsive subepithelial stromal layer of the lower genital tract. Typically, vulvar polyps are asymptomatic in their initial stages, and patients often delay seeking medical attention due to sociocultural factors. In this report, we present a case of a giant vulvar polyp and examine the underlying etiology and symptoms of this condition, highlighting the life stages of women that are most frequently affected. Additionally, we emphasize the rare but potential occurrence of malignant forms.

## Introduction

Fibroepithelial stromal polyps (FEPs) are commonly observed in women of reproductive age and typically manifest in the vagina, although they may also occur in the vulva and cervix with a lower frequency [1]. Soft tissue lesions in the distal female genital tract were first described in the early 1960s and are characterized as distinctive, benign stromal polyps [2]. FEPs in the lower female genital tract are benign growths, usually singular or less commonly multiple, characterized by the polypoid proliferation of stroma covered by squamous epithelium [3]. These lesions are hormone-sensitive and are commonly detected in women during their reproductive years, during pregnancy, or in premenopausal females taking hormone replacement therapy [4]. The incidence of fibroepithelial polyps, a benign mesenchymal tumor, is rare in prepubertal and postmenopausal individuals. Conversely, acrochordon, colloquially known as a skin tag, exhibits an age-related increase in prevalence and occurs in approximately a quarter of the adult population [5]. FEPs are one of several soft tissue lesions that may occur in the vulva, including cellular angiofibroma, angiomyofibroblastoma, superficial angiomyxoma, and aggressive angiomyxoma. An accurate diagnosis is necessary for appropriate treatment since these lesions exhibit distinct clinical behaviors. Additionally, routine hematoxylin and eosin slides may be used to identify morphological characteristics since these lesions may possess similar immunohistochemical and ultrastructural properties [3].

## Case presentation

**Figure 1:**
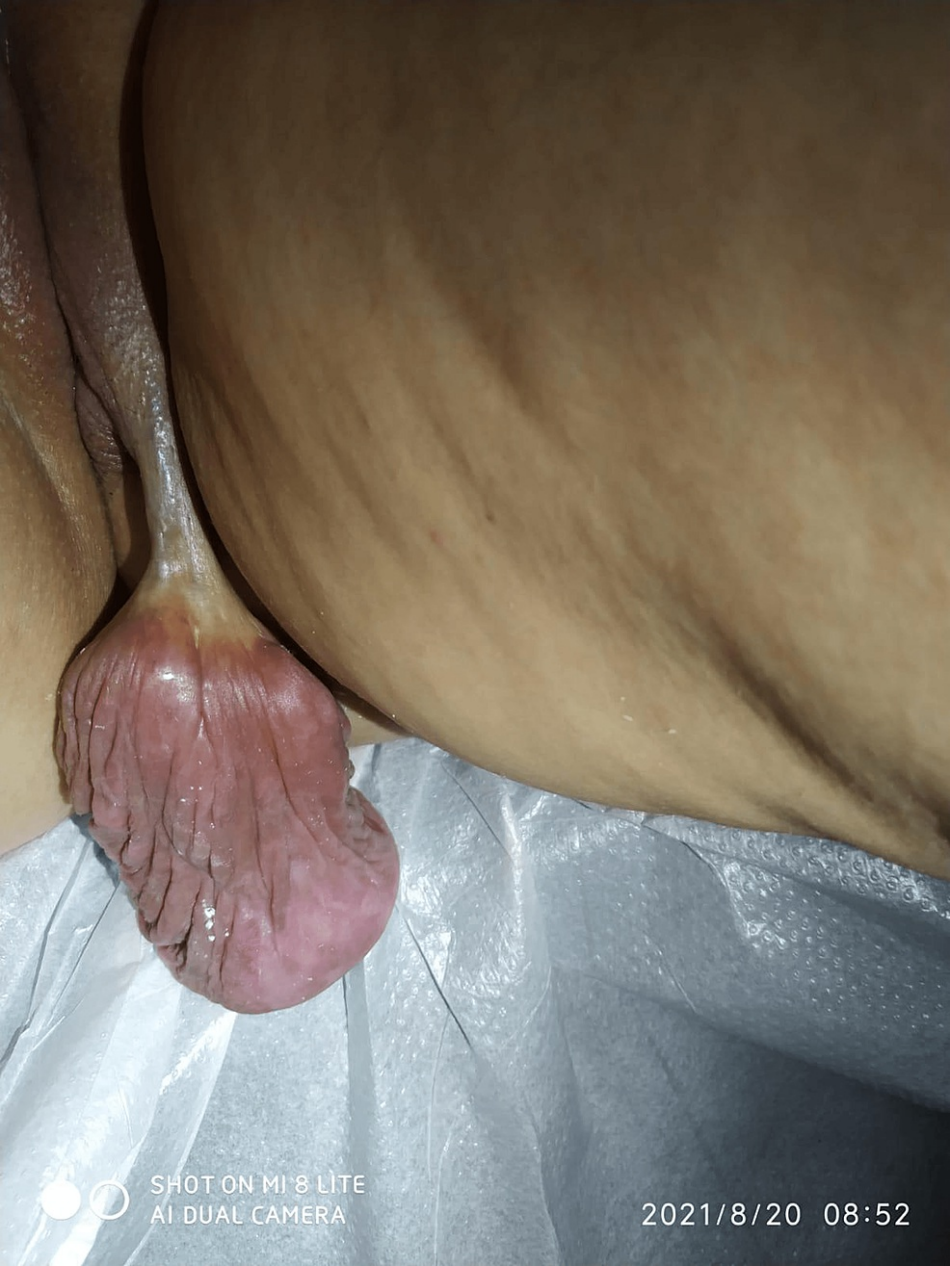
Physical examination of vulvar polyp

Upon presentation at our clinic, a 21-year-old female patient with a BMI of 22 presented with a mass located on the right vulva, which has been present for a duration of two years and exhibited recent growth over the past six months. In accordance with ethical guidelines governing the use of data and images, informed consent was obtained from the patient. The patient has refrained from engaging in sexual intercourse for a duration of two years, citing discomfort arising from the presence of a mass. Recently, the patient has reported a significant increase in the size of the aforementioned mass, further exacerbating the associated discomfort and cosmetic concerns. Physical examination revealed a painless mass of approximately 4 cm, originating from the inferior-posterior aspect of the right labium majus, extending to the thigh region, and measuring 10 cm in size. The mass was slightly firm to the touch and covered with normal skin, as depicted in Figure 1.

**Figure 2:**
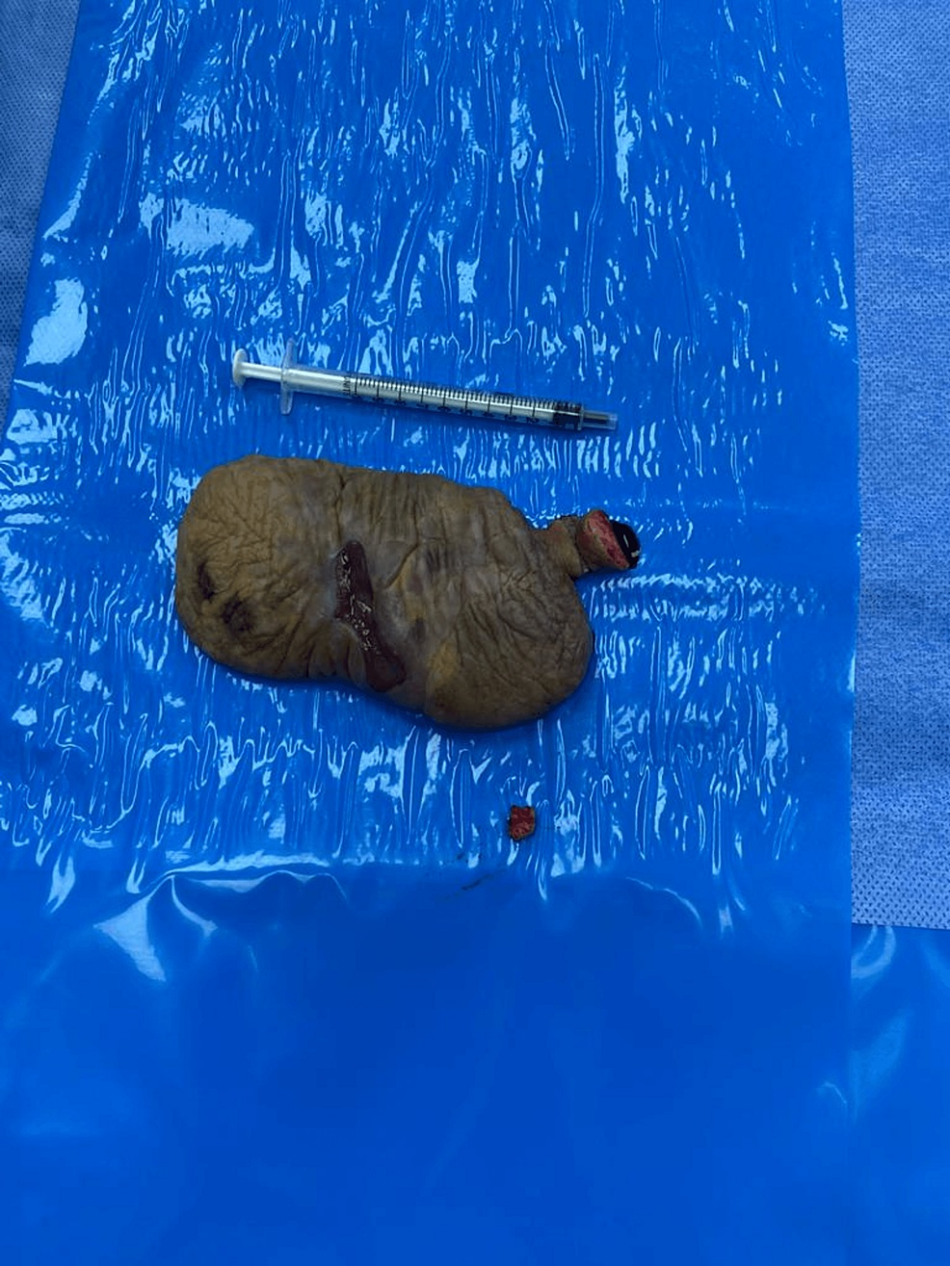
Image of vulvar polyp after excision

During the internal genital examination, no pathological findings were detected. Similarly, transvaginal ultrasonography revealed no abnormalities. The radiology department reported that the superficial ultrasonography results showed soft tissue elements. Given the location of the mass, both visual examination and superficial ultrasonography were used to rule out herniation. The patient was prepared for surgery, and under spinal anesthesia, excision of the mass and vulvar reconstruction were performed. The excision material exhibited a slightly firm consistency, measured 9 x 6 x 3 cm in size, and weighed approximately 100 g, as depicted in Figure 2.

**Figure 3:**
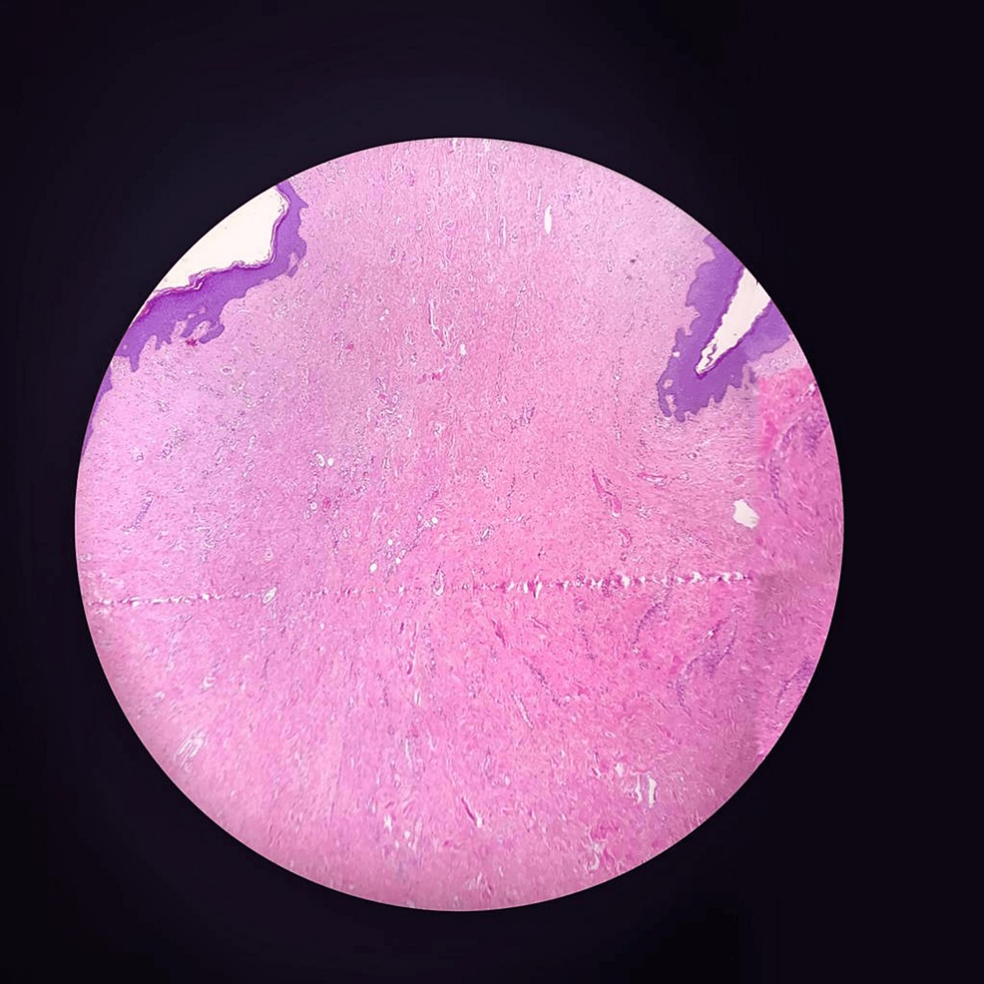
Desmin-positive ER

Upon pathological examination, a macroscopic observation revealed an ulcerated polypoid, a soft-consistent lesion on the surface. In the histopathological evaluation, a tissue covered with keratinized stratified squamous epithelium was observed on the surface, along with ulceration and granulation tissue in one area, and a hypocellular stroma lacking skin appendages. Immunohistochemical examination revealed desmin positivity for estrogen receptor (ER) (Figure 3).

**Figure 4:**
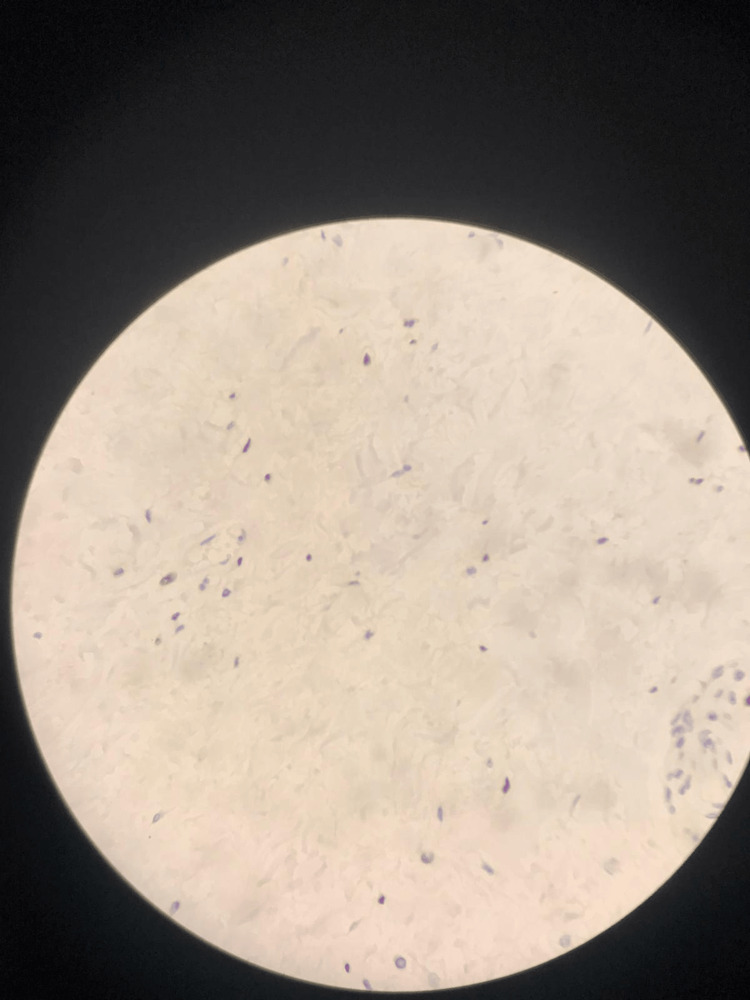
Hormone-sensitive stromal cells stained with ER in immunohistochemical examination

Based on these findings, the lesion was diagnosed as a fibroepithelial polyp, with stromal cells exhibiting sensitivity to hormones, as evidenced by ER staining (Figure 4).

## Discussion

Fibroepithelial polyps are benign lesions and may be an indicator of a hyperplastic process involving the subepithelial myxoid stroma, commonly mistaken for a malignant tumor. They are mostly small in size, the same color as the skin, and do not produce any symptoms [6]. They tend to grow, usually reaching a diameter of less than 5 cm. However, in rare cases, they can become remarkably large, sizing up to 15-20 cm [7].

These lesions are sensitive to hormones and frequently appear during pregnancy and at reproductive age. In about 15% of the cases identified, these polyps occur in pregnant women and are more often multiple. Upon delivery, they may regress spontaneously [8]. Moreover, premenopausal females taking hormone replacement therapy may also present these polyps [4]. They can have polypoid or pedunculated characteristics and are usually solitary. The only symptom experienced by our patient was a general discomfort with the sensation of a lump. Patients often have small asymptomatic lesions, but some of them may be associated with bleeding, discharge, or discomfort in accordance with the size and localization [9].

Even though it is uncommon, fibroepithelial polyps can reappear especially when not completely excised. There are several cases of such recurrence described in the literature [10].

In this report, we illustrate the case of a non-pregnant woman with a single FEP. The pathogenesis of FEP is not yet identified; however, some theories have been put forward. An important cause seems to be frequent irritation, particularly in women with obesity [11]. Hormone imbalances may also lead to the development of these lesions (e.g., high levels of estrogen and progesterone during pregnancy). Rarely do these stromal cells display marked atypia [1]. Therefore, it is sometimes confused with malignancies.

## Conclusions

FEPs are benign mesenchymal lesions that typically occur in women of reproductive age, although they can also be seen in prepubertal and postmenopausal individuals, albeit rarely. Hormonal changes during pregnancy can result in an increased incidence of multiple FEPs. Similarly, postmenopausal women receiving hormone replacement therapy may also have an elevated incidence of this condition. Although atypia can occur, it is important to be cautious in the differential diagnosis of malignancies. Microscopic evaluation of the polyp is critical for excluding malignancy.
